# Imbalance of Pro- vs. Anti-Coagulation Factors in Chinese Patients with Budd-Chiari Syndrome and Non-Cirrhotic Portal Vein Thrombosis

**DOI:** 10.1371/journal.pone.0119909

**Published:** 2015-03-30

**Authors:** Hui Chen, Lei Liu, Xingshun Qi, Chuangye He, Zhanxin Yin, Feifei Wu, Daiming Fan, Guohong Han

**Affiliations:** 1 Department of Liver Disease and Digestive Interventional Radiology, Xijing Hospital of Digestive Diseases, Xijing Hospital, Fourth Military Medical University, Xi’an, China; 2 State Key Laboratory of Cancer Biology and Xijing Hospital of Digestive Diseases, Xijing Hospital, Fourth Military Medical University, Xi’an, China; Northwestern University Feinberg School of Medicine, UNITED STATES

## Abstract

**Background and Aim:**

The coagulation abnormalities in non-cirrhotic Budd-Chiari syndrome (NC-BCS) and non-cirrhotic portal vein thrombosis (NC-PVT) are unclear. We conducted this case-control study to investigate the coagulation profile of NC-BCS and NC-PVT in Chinese patients.

**Methods:**

We measured the levels of factors II, V, VII, VIII, IX, X, XI, XII, protein C (PC), protein S (PS) and antithrombin (AT) in blood samples from 37 NC-BCS patients, 74 NC-PVT patients, and 100 healthy controls. The levels and ratios of pro- and anti-coagulation factors were compared between patients with NC-BCS and healthy controls, between different types of NC-BCS and between NC-PVT and healthy controls.

**Results:**

In patients with NC-BCS, factor VIII (P<0.001) was significantly elevated; factor V (P<0.001), VII (P<0.001), IX (P = 0.003), X (P<0.001), XI (P<0.001), XII (P<0.001), PC (P<0.001) and AT (P<0.001) were significantly decreased; and no difference was observed for factor II (P = 0.088) and PS (P = 0.199) compared with healthy controls. Factor VIII-to-PC (P = 0.008), factor VIII-to-PS (P = 0.037) and factor VIII-to-AT (P = 0.001) were significantly increased; other ratios were significantly reduced or did not show any difference. No differences were observed between different types of NC-BCS for individual pro- and anti-coagulation factors or the ratios between them. Among patients with NC-PVT, factor VIII (P<0.001) was significantly elevated and other factors were significantly decreased. Factor II-to-PC (P<0.001), factor VIII-to-PC (P<0.001), factor IX-to-PC (P<0.001), factor VIII-to-PS (P<0.001), factor II-to-AT (P<0.001), factor VIII-to-AT (P<0.001) and factor IX-to-AT (P<0.001) were significantly increased; all other ratios for NC-PVT were significantly reduced or did not show any significant difference.

**Conclusions:**

NC-BCS and NC-PVT are associated with elevated levels of factor VIII and the decreased levels of PC and AT were probably the most significant features of coagulation imbalance. Additionally, NC-PVT was associated with decreased levels of PS.

## Introduction

Coagulation is a highly integrated cellular and humoral process that is balanced by two opposing factors [1]. The primary pro-coagulation factors include factor II, factor V, factor VII, factor VIII, factor IX, factor X, factor XI, and factor XII; and the primary anti-coagulation factors include protein C (PC), protein S (PS), and antithrombin (AT). Most pro- and anti-coagulation factors, with the exception of factor VIII, are synthesized by hepatocytes [2]. The mechanism of clot formation is dependent on the interaction of pro- and anti-coagulation factors [3]. The balance between these factors is crucial for normal hemostasis, and the disturbances of this balance may lead to a thrombotic tendency [4]. Furthermore, the ratios between the levels of the individual pro- and anti-coagulants can be taken as indexes of the coagulation imbalance (the greater the ratios, the higher the procoagulant imbalance) [1, 3].

Budd-Chiari syndrome (BCS) and portal vein thrombosis (PVT) are two major vascular disorders of the liver characterized by obstruction of the hepatic and the portal vein system [5]. The portal venous system directs blood from the abdominal gastrointestinal tract, spleen, pancreas, and gallbladder to liver and the hepatic venous outflow travels through the three hepatic veins to the inferior vena cava [6]. Primary BCS and PVT are rare but life-threatening vascular disorders of the liver that can result in portal hypertension [5, 6]. There is a considerable overlap in the etiology of patients with BCS and PVT, which are closely associated with underlying inherited or acquired thrombotic risk factors. The inherited thrombophilia mainly includes factor V Leiden (FVL) G1691A mutation, prothrombin G20210A polymorphism [7–9], and the inherited deficiencies of PC, PS or AT [9–11]. A recent systematic review and meta-analysis revealed that the presence of the FVL mutation may increase the risk of BCS and non-cirrhotic PVT (NC-PVT); the presence of the prothrombin mutation is associated with an increased risk of NC-PVT, but not with BCS [12]. In addition, studies have revealed that 80% of patients have at least one thrombotic risk factor, and 30%-50% of patients have a combination of these risk factors [13, 14].

However, the etiological distribution of these diseases might be different between Western and Chinese patients. An observational study from our center and a systematic review of the literature revealed that FVL mutation and prothrombin G20210A mutation are rarely found in Chinese BCS patients [15]. Meta-analysis also revealed that inherited PC, PS and AT deficiencies are rare in BCS and NC-PVT patients both from Europe and Asia [16]. Furthermore, there is scant research to investigate coagulation profile and the imbalance between pro- and anti-coagulation factors in Chinese patients with these two disorders and evidence from existing studies is conflicting. A study by Raffa et al. thoroughly investigated the coagulation system of patients with NC-PVT and found that patients with NC-PVT had hypercoagulability that is independent of the underlying etiology [17]. However, in the study of Chaireti et al. no significant difference of hypercoagulability was observed between BCS and NC-PVT patients and their respective controls [18].

Therefore, we conducted this case-control study to thoroughly investigate the plasma levels of pro- and anti-coagulant factors, as well as the ratios between them, in BCS patients without cirrhosis (NC-BCS) and those with NC-PVT.

## Patients and Methods

### Ethics Statement

The study protocol conformed to the ethical guidelines of the 1975 Declaration of Helsinki and was approved by the ethics committee of Xijing Hospital. Written informed consent was obtained from each patient.

### Study population

Between August 2011 and March 2014, all the patients referred to our hospital with a diagnosis of primary NC-BCS or NC-PVT were screened for eligibility to participate in the present case-control study. Notably, because the levels of pro- and anti-coagulation factors could be largely influenced by liver function, the presence of liver cirrhosis was clearly excluded from our study population based on clinical, laboratory and imaging studies or liver biopsy. The exclusion criteria were as follows: BCS with liver cirrhosis, PVT with liver cirrhosis, hepatocellular carcinoma or other cancer, splenectomy, known hemostatic disorders other than cirrhosis and blood transfusion within 3 days, and receipt of percutaneous transluminal angioplasty or stent placement. A total of 37 consecutive patients with NC-BCS and 74 patients with NC-PVT were enrolled in this study. An additional 100 healthy non-relatives of matched age, gender, and ethnic background were enrolled as controls for patients with NC-BCS and those with NC-PVT. All controls had no history of previous thrombosis.

### Measurements

The levels of pro- and anti-coagulation factors were measured as previously reported with results expressed as the percentage of a normal pooled plasma, which was set at 100%[3]. Patients receiving anti-platelet agents or anticoagulants were required to cease the drugs at least 2 weeks before enrollment. Fasting peripheral blood samples were taken from the antecubital vein in a standardized manner. Blood was drawn (3 ml) into vacuum tubes (Improve Medical, Guangzhou, China) containing 3.8% sodium citrate (ratio 9:1) as an anti-coagulant. Blood was centrifuged at 3000 g for 25 minutes at 18°C within 30 minutes of collection. Fresh platelet-poor plasma was then harvested, and all coagulation tests were performed within 4 hours after the blood was drawn. All hemostasis tests were performed in an automated blood coagulation analyzer (CA-7000, Sysmex, Hyogo, Japan) using standard reagents. The activities of coagulation factors II, V, VII, VIII, IX, X, XI, and XII were determined by one-stage clotting assays using factor-deficient plasmas (Siemens, Marburg, Germany). The activities of PC and AT were measured using chromogenic assays (Berichrom PC, Berichrom AT, Siemens, Marburg, Germany). The activity of PS was also measured by a clotting assay (PS Ac, Siemens, Marburg, Germany).

### Definitions

All included patients underwent color Doppler ultrasound as well as abdominal contrast-enhanced computed tomography or magnetic resonance imaging for the evaluation of the inferior vena cava (IVC), hepatic vein (HV) and portal venous system.

Primary BCS was diagnosed according to previously published criteria [19] and NC-BCS was identified according to the absence of pre-existing cirrhosis. Moreover, according to the obstructive location, we divided NC-BCS into three types as previously described [20, 21]: i) IVC-type, manifested as obstruction of the IVC with at least one HV; ii) HV-type, manifested as obstruction of the three main HVs; and iii) combined-type, manifested as an obstruction of both the IVC and the three main HVs.

NC-PVT is characterized by the formation of a thrombus within the portal vein trunk and intrahepatic portal branches in the absence of liver cirrhosis. Portal cavernoma detected in the form of a mass-like network of numerous hepatopetal collateral vessels located in the liver hilum defines patients having chronic PVT[22].

Inherited PC, PS or AT deficiency was assumed if any one of the following criteria was met: (i) the deficiency was present in at least one first- or second-degree family member (family study), (ii) a causative gene mutation was identified (genetic analysis)[16], or (iii) a ratio of PC, PS or AT to (factor II + factor X)/2 below 0.7 indicated the need for evaluation of the possible presence of hereditary protein deficiencies[9, 23, 24].

### Statistical Analysis

Continuous variables were expressed as mean and standard deviation (SD), and categorical variables were presented as frequencies. The chi-square test was used to compare categoric variables between patients and controls and the Student t test, analysis of variance, or the Mann–Whitney U test were used as appropriate for continuous variables. Two-tailed p values less than 0.05 were considered significant. All data were analyzed using SPSS 16.0 software (SPSS Inc., Chicago, IL).

## Results

Baseline demographic and clinical characteristics of the included patients with NC-BCS and NC-PVT at admission are shown in Table 1. The mean age (±SD) age was 37.7±12.1 years and 38.6±15.8 years for NC-BCS and NC-PVT, respectively. Gastrointestinal bleeding manifest as hematemesis or melena was observed in 3 (8.1%) and 39 (52.7%) patients in NC-BCS and NC-PVT group, respectively. For patients with NC-PVT, an inherited deficiency of PC, PS or AT was observed in 2, 1 and 1 patients, respectively. Another 6 patients had combined deficiencies of PC, PS or AT.

**Table 1:** Demographic characteristics of patients with NC-BCS and NC-PVT.

Parameters	NC-BCS (n = 37)	NC-PVT (n = 74)
Age, years	37.7±12.1	38.6±15.8
Gender, male/female, n	25/12	40/34
Location of BCS		
IVC-type, n (%)	8 (21.6)	/
HV-type, n (&)	13 (35.1)	/
combined-type, n (%)	16 (43.2)	/
Location of PVT		
IHPV, n (%)	/	42 (56.7)
MPV, n (%)	/	74 (100)
SV, n (%)	/	22 (29.7)
SMV, n (%)	/	28 (37.8)
Portal cavernoma, n (%)	/	47 (63.5%)
Clinical manifestations		
Abdominal pain, n (%)	9 (24.3)	27 (36.5)
Lower-extremity edema, n (%)	11 (29.7)	2 (2.7)
Hematemesis/melena, n (%)	3 (8.1)	39 (52.7)
Albumin, mg/dl	40 ± 7.2	38 ±5.6
Bilirubin, μmol/L	26.3 ± 16.4	21.2 ± 15.4
Creatinine, mg/dl	80.5 ± 15	76.7 ± 16.8
Leukocytes, x 10^9^/L	4.8 ± 2.1	5.34 ± 4.7
Hemoglobin, g/L	130 ± 24	95 ± 31.4
Platelets, x10^9^/L	109.3 ± 50	139.6 ± 119.8
PT, s	14.6 ± 1.6	15.7 ± 2.3
INR	1.2 ± 0.2	1.28 ± 0.23
Serum sodium, mmol/L	141.2 ± 2.4	140 ± 2.4

**NOTE.** Values are expressed as mean ± SD; n indicates number of cases. **Abbreviations:** HV, hepatic vein; IHPV, intrahepatic portal vein; INR, international normalized ratio; IVC, inferior vena cava; MELD, Model for End-stage Liver Disease; MPV, main portal vein; NC-BCS, non-cirrhotic non-tumoral Budd-Chiari Syndrome; NC-PVT, non-cirrhotic non-tumoral portal vein thrombosis; PT, prothrombin time; SMV, superior mesenteric vein; SV, splenic vein.

### Comparison between patients with NC-BCS and healthy controls

**Fig 1:**
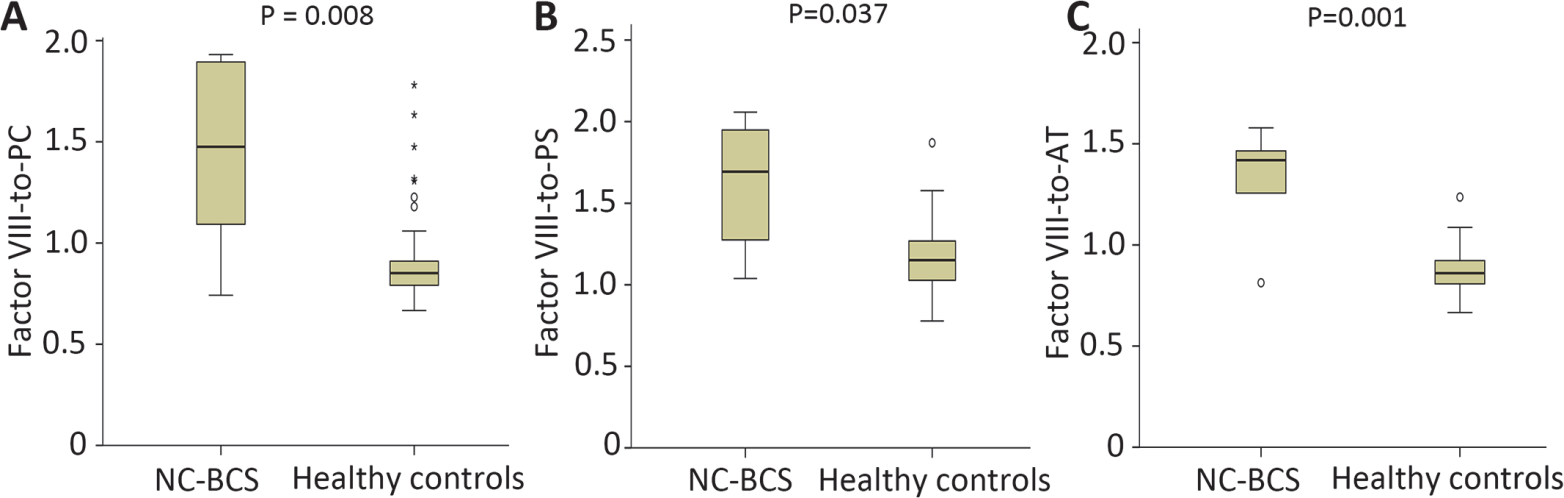
Box plots for the ratios of factor VIII-to-PC, factor VIII-to-PS, and factor VIII-to-AT between patients with non-cirrhotic Budd-Chiari syndrome (NC-BCS) and healthy controls.

The pro- and anti-coagulation factors for patients with NC-BCS are reported in Table 2. Compared with healthy controls, factor VIII (P<0.001) was significantly elevated; factor V (P<0.001), VII (P<0.001), IX (P = 0.003), X (P<0.001), XI (P<0.001), XII (P<0.001), PC (P<0.001) and AT (P<0.001) were significantly decreased; and no difference was observed for factor II (P = 0.088) and PS (P = 0.199) in NC-BCS patients. The ratios between pro- and anti-coagulation factors for patients with NC-BCS are reported in Table 3. Compared with healthy controls, the factor VIII-to-PC (P = 0.008), factor VIII-to-PS (P = 0.037) and factor VIII-to-AT (P = 0.001) ratios were significantly increased (Fig. 1.); factor V-to-PS (P = 0.006), factor VII-to-PC (P = 0.031), factor VII-to-PS (P<0.001), factor VII-to-AT (P = 0.017), factor X-to-PS (P<0.001), factor X-to-AT (P = 0.047), factor XI-to-PS (P<0.001), factor XI-to-AT (P = 0.001), factor XII-to-PS (P<0.001), and factor XII-to-AT (P = 0.031) were significantly reduced in NC-BCS patients. All other ratios of pro- and anti-coagulation factors did not show any significant difference between the two groups (Table 3). Besides, no significant correlation between coagulation factors and liver function parameter was found for these patients (Table 4).

**Table 2:** Pro- and anti-coagulation factors for patients with NC-BCS, NC-PVT and healthy controls.

Variables	NC-BCS (n = 37)	NC-PVT (n = 74)	Healthy (n = 100)
Factor II, %	***86.7± 20.2***	82.7± 21.8	91.6±16.4
Factor V, %	78.7± 27.1	72.8± 31.3	103.9±8.2
Factor VII, %	70.2± 30.7	76.1± 26.8	103.5±10.3
Factor VIII, %	139 ± 65.6	157 ± 74.7	84.1 ± 8.6
Factor IX, %	75.6 ± 19.2	77.6 ± 26.2	83.9 ± 13.2
Factor X, %	78.8 ± 19.5	68.1 ± 21.5	98.2 ± 6.8
Factor XI, %	64 ± 14.9	67.1 ± 30	87.3 ± 9.6
Factor XII, %	61.3 ± 25.9	53 ± 23	84 ± 8.5
PC, %	76.1 ± 18.3	71.2 ± 25	96.6 ± 9.9
PS, %	***76.9 ± 16.2***	66.1 ± 23	72.3 ± 1.5
AT, %	86.9 ± 14.7	74.6 ± 18.5	97.9 ± 5.1

**NOTE.** Values are expressed as mean ± SD. Bold Italic value indicates P > 0.05 when compared with the control group.

**Abbreviations:** AT, antithrombin; NC-BCS, non-cirrhotic non-tumoral Budd-Chiari Syndrome; NC-PVT, non-cirrhotic non-tumoral portal vein thrombosis; PC, protein C; PS, protein S.

**Table 3:** Ratios between pro- and anti-coagulant factors for patients with NC-BCS, NC-PVT and healthy controls.

Ratios	NC-BCS (n = 37)	NC-PVT (n = 74)	Controls (n = 100)
Factor II /PC	1.14 ± 0.28	1.22 ± 0.37*	0.96 ± 0.24
Factor V /PC	1.12 ± 0.22	1.02 ± 0.36	1.09 ± 0.19
Factor VII /PC	0.83 ± 0.15*	1.15 ± 0.41	1.09 ± 0.20
Factor VIII /PC	1.86 ± 1.19*	2.22 ± 1.10*	0.88 ± 0.18
Factor IX /PC	1.03 ± 0.38	1.12 ± 0.34*	0.88 ± 0.21
Factor X /PC	1.01 ± 0.26	1.01 ± 0.26	1.03 ± 0.16
Factor XI /PC	0.79 ± 0.18	0.87 ± 0.21	0.92 ± 0.17
Factor XII /PC	0.72 ± 0.18	0.82 ± 0.33	0.89 ± 0.19
Factor II /PS	1.17 ± 0.24	1.33 ± 0.45	1.38 ± 0.73
Factor V /PS	1.18 ± 0.36*	1.05 ± 0.45*	1.56 ± 0.79
Factor VII /PS	0.84 ± 0.33*	1.17 ± 0.29*	1.56 ± 0.81
Factor VIII /PS	1.89 ± 0.82*	2.18 ± 0.74*	1.28 ± 0.77
Factor IX /PS	1.07 ± 0.32	1.17 ± 0.33	1.29 ± 0.95
Factor X /PS	0.99 ± 0.16*	1.04 ± 0.25*	1.47 ± 0.74
Factor XI /PS	0.83 ± 0.22*	0.93 ± 0.26*	1.33 ± 0.86
Factor XII /PS	0.76 ± 0.19*	0.84 ± 0.34*	1.27 ± 0.72
Factor II /AT	1.02 ± 0.14	1.15 ± 0.26*	0.94 ± 0.19
Factor V /AT	1.02 ± 0.21	1.06 ± 0.10	1.02 ± 0.48
Factor VII /AT	0.76 ± 0.17*	1.05 ± 0.32	1.05 ± 0.10
Factor VIII /AT	1.57 ± 0.70*	2.05 ± 0.64*	0.86 ± 0.09
Factor IX /AT	0.90 ± 0.16	1.09 ± 0.37*	0.86 ± 0.15
Factor X /AT	0.88 ± 0.11*	0.98 ± 0.33	1.00 ± 0.08
Factor XI /AT	0.71 ± 0.13*	0.87 ± 0.28	0.89 ± 0.10
Factor XII /AT	0.66 ± 0.19*	0.82 ± 0.41	0.86 ± 0.09

**NOTE.** Values are expressed as median (range).

* indicates P < 0.05 when compared with the healthy controls.

**Abbreviations:** AT, antithrombin; NC-BCS, non-cirrhotic non-tumoral Budd-Chiari syndrome; NC-PVT, non-cirrhotic non-tumoral portal vein thrombosis; PC, protein C; PS, protein S.

**Table 4:** The P values of correlation between pro- and anti-coagulation factors with liver function parameters (albumin and bilirubin) for patients with NC-BCS and NC-PVT.

Variables	NC-BCS (n = 37)		NC-PVT (n = 74)	
	Albumin	Bilirubin	Albumin	Bilirubin
Factor II	0.611	0.148	0.271	0.609
Factor V	0.433	0.556	0.746	0.081
Factor VII	0.525	0.120	0.256	0.513
Factor VIII	0.096	0.724	0.463	0.402
Factor IX	0.757	0.141	0.593	0.473
Factor X	0.371	0.253	0.060	0.286
Factor XI	0.586	0.386	0.243	0.093
Factor XII	0.188	0.740	0.101	0.558
PC	0.263	0.440	0.081	0.097
PS	0.112	0.031	0.275	0.380
AT	0.258	0.701	0.274	0.973

**Abbreviations:** PC, protein C; PS, protein S; AT, antithrombin; NC-BCS, non-cirrhotic non-tumoral Budd-Chiari Syndrome; NC-PVT, non-cirrhotic non-tumoral portal vein thrombosis.

### Comparison between different types of NC-BCS

We compared the plasma levels of all pro- and anti-coagulation factors between different types of BCS, and no differences were observed. Additionally, no differences in the ratios between pro- and anti-coagulation factors were found between different types of NC-BCS.

### Comparison between NC-PVT and healthy controls

**Fig 2:**
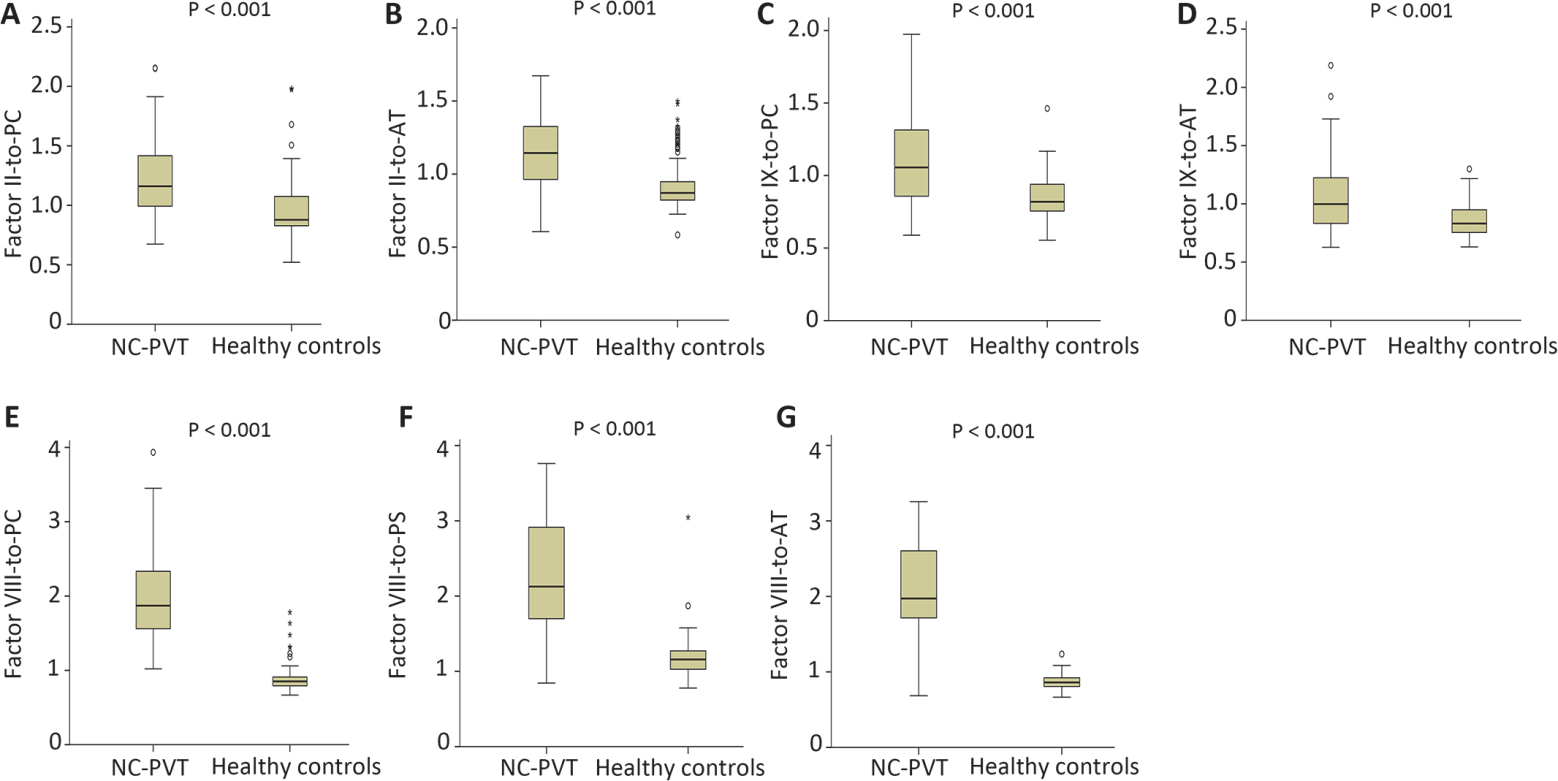
Box plots for the increased ratios in patients with non-cirrhotic portal vein thrombosis (NC-PVT) and healthy controls.

In patients with NC-PVT, factor VIII was significantly elevated (P<0.001) and factors II (P = 0.007), V (P<0.001), VII (P<0.001), IX (P = 0.035), X (P<0.001), XI (P<0.001), XII (P<0.001), PC (P<0.001), PS (P = 0.044) and AT (P<0.001) were significantly decreased when compared with healthy controls (Table 2). Among all of the ratios between pro- and anti-coagulation factors in patients with NC-PVT (Table 3), factor II-to-PC (P<0.001), factor VIII-to-PC (P<0.001), factor IX-to-PC (P<0.001), factor VIII-to-PS (P<0.001), factor II-to-AT (P<0.001), factor VIII-to-AT (P<0.001) and factor IX-to-AT (P<0.001) were significantly increased (Fig. 2); factor V-to-PS (P<0.001), factor VII-to-PS (P = 0.006), factor X-to-PS (P = 0.001) factor XII-to-PS (P = 0.008) and factor XII-to-PS (P = 0.001) were significantly reduced; and all other ratios did not show any difference between the two groups (Table 3). Additionally, no significant correlation between coagulation factors and liver function parameter was found for patients with NC-PVT (Table 4). To exclude the confounding bias of hematemesis/melena, we compared the patients presenting with hemorrhage with those without hemorrhage and no differences in pro- and anti-coagulation factors were observed (Table 5).

**Table 5:** Pro- and anti-coagulation factors for patients with and without hemorrhage in the NC-PVT group.

Variables	Hemorrhage (n = 39)	Non-hemorrhage (n = 35)	P value
Factor II, %	81.1 ± 16.2	84.9 ± 28.2	0.588
Factor V, %	70.2± 30.4	76.4 ± 32.9	0.506
Factor VII, %	72.7± 20.1	80.7 ± 33.9	0.353
Factor VIII, %	152.7 ± 73.1	163.1 ± 78.4	0.647
Factor IX, %	71.1 ± 19.5	87.1 ± 31.6	0.060
Factor X, %	63.7 ± 16.9	74.2 ± 25.9	0.124
Factor XI, %	65.1 ± 32.7	69.8 ± 26.3	0.584
Factor XII, %	52.5 ± 20.6	53.7 ± 28.8	0.858
PC, %	69.1 ± 20.8	74.2 ± 30.5	0.486
PS, %	64.5 ± 17.6	68.6 ± 29.6	0.555
AT, %	72.9 ± 16.0	77.2 ± 21.6	0.398

**NOTE.** Values are expressed as mean ± SD.

**Abbreviations:** AT, antithrombin; PC, protein C; PS, protein S; NC-PVT, non-cirrhotic non-tumoral portal vein thrombosis.

## Discussion

Recently, our understanding of coagulation imbalance in patients with chronic liver disease has changed greatly and a pro-coagulant imbalance associated with chronic liver disease can be detected by measuring thrombin generation [4]. It was hypothesized that the pro-coagulant imbalance might be due to two combined abnormalities: low levels of protein C and high plasma levels of factor VIII [1]. Similar changes have been observed in patients with non-alcoholic fatty liver disease [25]. What is more, by in vitro addition of protein C, the pro-coagulant imbalance is considerably reduced indicating that low protein C contributes to the procoagulant imbalance in plasma from patients with cirrhosis [26]. These findings encouraged us to investigate the role of coagulation imbalance in the development of BCS and NC-PVT.

BCS and NC-PVT are two entities of splanchnic vein thrombosis. BCS is defined as hepatic venous outflow obstruction at any level from the small hepatic veins to the junction of the inferior vena cava and the right atrium, regardless of the cause of obstruction [27]. PVT refers to the complete or partial obstruction of blood flow in the portal vein, due to the presence of a ‘thrombus’ in the vessel lumen [28]. In both disorders, pathogenesis are very complex [19, 24, 29], and the occurrence of the two diseases can be influenced by both local and systemic factors; several prothrombotic, acquired and heritable factors interact to cause thrombosis in these uncommon locations [9, 13, 30]. Currently, the contribution of coagulation imbalance to the pathogenesis and the coagulation profile of BCS and NC-PVT have not been fully established, especially in Chinese patients. To exclude the interference of liver function damage, the patients with liver cirrhosis were excluded from the study. Additionally, we did not observe any significant correlation between coagulation factors and liver function was observed.

## Coagulation Imbalance in Patients with NC-BCS

There are little data about the role of haemostatic imbalance in the development of BCS due to the rarity of the condition. Moreover, most studies on the pathogenesis of BCS are hampered by the absence of a well-documented control groups. A high level of factor VIII has been recognized as a risk factor for venous thromboembolism [31] and Koshy et al. reported 2 BCS patients with familial high factor VIII level [32]. Recently, thrombin generation had been a considered as a marker for hypercoagulability but in Chaireti’s study, thrombin generation in patients with BCS did not differ from that in healthy controls [18]. In the present study, we found a global reduction of pro- and anti-coagulation factors except for factor VIII, which was elevated, and factor II and PS, which were not altered. These changes were similar to the abnormalities observed in patients with chronic liver disease [1, 4, 25, 26]. But we do not have a suitable explanation for the levels of factor II and PS. A trend for decrease was observed for factor II (P = 0.088) and a significant difference may be found in a larger sample size. In the study, we measured the activity of PS which might be easily affected by the increased levels of factor VIII and other perturbations of the coagulation cascade. Free PS antigen is considered by many to be a more reliable assay but was not measured in the present study [33]. In addition, factor VIII-to-PC, factor VIII-to-PS and factor VIII-to-AT ratios were significantly increased. Considering the activity of PS was similar between two groups, it can be inferred that for NC-BCS, the elevated levels of factor VIII and the decreased levels of PC and AT may be the most significant features of coagulation imbalance which may play an important role in the pathogenesis of NC-BCS. The role of PS need further clarify. The relatively low number of observations in the BCS group limits the conclusions and this result needs further larger studies to clarify.

## Coagulation Imbalance in Patients with NC-PVT

Two studies have investigated the association between coagulation system and NC-PVT [17, 18]. But these two studies obtained contradictory results with regard to the thrombin generation test. Additionally, some authors also found that high factor VIII levels were associated with NC-PVT [34, 35]. For the pro- and anti-coagulation imbalance, both the present study and Raffa’s study documented a global reduction of pro- and anti-coagulation factors except for factor VIII. These changes are similar to the coagulation changes in chronic liver disease. In the present study, no relationship was found between alteration of coagulation parameters and parameters of liver function. These changes may be due to an increased clearance or consumption caused by the presence of portal hypertension or portosystemic shunting [17]. The increased ratios of factor II-to-PC, factor II-to-AT, factor IX-to-PC and factor IX-to-AT were close to one while the mean ratios of factor VIII-to-PC, factor VIII-to-PS and factor VIII-to-AT were above 2. So we can infer that NC-PVT were characterized by the elevated levels of factor VIII and the decreased levels of PC, PS and AT and may play an important role in the pathogenesis of NC-PVT.

### Coagulation imbalance and NC-BCS type

IVC obstruction with or without HV involvement is predominant in Asian populations, while pure HV obstruction is reported more often in Western countries [36]. Moreover, comprehensive investigations have shown similar underlying thrombophilias for both sites, in agreement with the results of the present study [37]. Regardless of the pro- and anti-coagulation factors, or the ratios between them, no difference between the different types of BCS was observed. We can thus infer that the coagulation abnormalities may not be the reason for the BCS different types. What remains to be elucidated is why the IVC or HV is so frequently involved in Asian or Western populations, respectively, and the underlying causes.

### Limitations

A potential limitation of the present study is the in vitro ratios of pro- and anti-coagulant factors are not truly representative of their in vivo expression, but their changes can roughly reflect the imbalance of coagulation. Another limitation was the relatively small number of patients in the subgroup analysis of NC-BCS types.

In conclusion, for NC-BCS, the elevated levels of factor VIII and the decreased levels of PC and AT may be the most important changes of coagulation system and may play an important role in the pathogenesis. The role of PS needs further clarification. For NC-PVT, the elevated levels of factor VIII and the decreased levels of PC, PS and AT may play an important role in the pathogenesis. Coagulation imbalance may not play a role in the formation of different types of BCS. Additional studies are warranted using more reliable methods, such as thrombin generation assays, to assess the global profile of coagulation system in vivo for NC-BCS and NC-PVT.
